# Molecular epidemiology of *Mycoplasma hyorhinis* porcine field isolates in the United States

**DOI:** 10.1371/journal.pone.0223653

**Published:** 2019-10-21

**Authors:** Maria J. Clavijo, Srinand Sreevatsan, Timothy J. Johnson, Albert Rovira

**Affiliations:** 1 Department of Veterinary Population Medicine, College of Veterinary Medicine, University of Minnesota, St Paul, Minnesota, United States of America; 2 Department of Veterinary and Biomedical Sciences, University of Minnesota, St Paul, Minnesota, United States of America; Defense Threat Reduction Agency, UNITED STATES

## Abstract

*Mycoplasma hyorhinis* is one of the causative agents of polyserositis and arthritis in post-weaning pigs. Here we describe the development of a multi-locus sequence typing (MLST) protocol for the characterization of *M*. *hyorhinis* field isolates. A total of 104 field isolates from different geographical locations, swine production systems, and clinical backgrounds, were analyzed. Twenty-seven genes, including housekeeping and those encoding surface proteins, were evaluated to index diversity. Genes encoding surface proteins were included to increase the discriminatory power of the MLST. Four target gene fragments were selected to be included in the final MLST-s (surface) protocol: *pdhB*, *p95*, *mtlD* and *ung*. Within each locus the nucleotide variation ranged from 1.4% to 20%. The 104 field isolates were classified into 39 distinct sequence types (STs). Multiple STs were found within the same production system and within the same pig. The majority of STs grouped strains from the same production system; however, cases existed where multiple systems shared a ST, indicating potential relationships between pig flows. The majority of the nucleotide changes observed in these genes generated synonymous changes, while non-synonymous changes were exclusively in the *mtlD* gene fragment, suggesting that this protein is undergoing selection. Molecular typing of *M*. *hyorhinis* will primarily aid swine practitioners with pig flow management and identifying sources of infection during outbreaks.

## Introduction

In recent years, *Mycoplasma hyorhinis* has been recognized as an important cause of mortality in nursery pigs [1,2]. This pathogen colonizes the nasal and oropharyngeal epithelial surface of pigs. Piglets presumably become colonized through contact with sows, and the bacterium is transmitted via nose-to-nose contact among pigs following colonization and infection [3]. Although *M*. *hyorhinis* infections are frequently subclinical, they can cause polyserositis and arthritis in post-weaning pigs. In fact, 50% of polyserositis and approximately 10% of arthritis cases received annually at the Minnesota Veterinary Diagnostic Laboratory test positive for this pathogen by a Polymerase Chain Reaction (PCR)-based diagnostic testing method (Personal observations). *M*. *hyorhinis*-associated disease in finishing pigs is usually characterized by arthritis alone [4]. Other clinical presentations, including pneumonia, otitis, and conjunctivitis, have been reported [5,6]. However, the pathogenic significance of *M*. *hyorhinis* in these disease presentations is uncertain.

Evidence of heterogeneity between isolates within the *M*. *hyorhinis* species exists, including antigenic differences evidenced by seroreactivity to specific antisera [7,8], differences in virulence using *in vivo* models of infection [5, 9–11], and variable clinical presentation of disease between herds. While some herds are repeatedly affected by *M*. *hyorhinis-*associated arthritis, others tend to exhibit mostly polyserositis [12]. This diverse disease presentation also suggests that strains with different tissue tropisms may exist.

Molecular epidemiological techniques allow for the identification of sources of infection, tracking the spread of infectious microorganisms, and evaluating persistence or reintroduction of a pathogen in a population. Moreover, molecular epidemiology can play an important role in disease control, since molecular methods can be used to determine potential vaccine targets and predict vaccine efficacy [13, 14]. At the genome level, the use of pulsed-field gel electrophoresis (PFGE) to index variation within the *M*. *hyorhinis* species has revealed genetic diversity within the species[15,16]. ]PFGE requires relatively pure cultures and is time consuming, however, and interpretation of the resulting banding patterns can be ambiguous in some cases. Furthermore, since it identifies major genetic changes, small DNA changes will be missed [17]. Sequence-based tools, such as MLST (multilocus sequence typing), have been widely used for genotyping bacteria causing infections in pigs [18–22]. Furthermore, MLST is considered a rapid, inexpensive, repeatable, and unambiguous typing tool [23]. A traditional MLST scheme, based on the *dnaA*, *rpoB*, *gyrB*, *gltX*, *adk* and *gmk* genes, has been developed for *M*. *hyorhinis* [18, 23]. The variation reported by the authors showed a low percentage of polymorphic sites, ranging from 0.9–2.1%. A recently optimized version of the same MLST scheme evaluated the genetic diversity of 60 strains from Germany and Switzerland. Similarly, the authors reported genetic variation between strains with limited clonality [22]. While core metabolic genes are ideal for inferring phylogenic relationships, they can lack discriminatory power for differentiation of closely related strains [24]. In this manuscript, we describe the development of a novel MLST assay based on housekeeping and surface-encoding proteins to differentiate *M*. *hyorhinis* strains, termed MLST-s to reference the addition of surface proteins. Surface proteins were added to increase discriminatory power, since these genes may be subject to more intensive selective pressures compared to housekeeping genes, making them ideal for local epidemiological studies [24].

## Materials and methods

### Bacterial isolates, culture media and DNA extraction

A total of 104 *M*. *hyorhinis* isolates from pigs along with the *M*. *hyorhinis* type strain ATCC 17981 were studied (S1 Table). Isolates originated from the nasal cavity (n = 3), bronchus (n = 26), pericardium (n = 26), pleura (n = 21), peritoneum (n = 2) and joint (n = 19) of diseased pigs with lesions of polyserositis and a clinical history suggestive of *M*. *hyorhinis* infection (fever, depression, lameness, dyspnea). Six isolates originated from the bronchus (n = 1) and nasal cavity (n = 5) of healthy pigs. Finally, one isolate was obtained from an air sample taken from the outside of a barn housing nursery pigs. All isolates were obtained from cases submitted to the UMN Veterinary Diagnostic Laboratory from 2010 to 2012. All isolates were confirmed as *M*. *hyorhinis* utilizing a qPCR assay based on the 16S rRNA gene [25]. The isolates originated from multiple farms (nurseries, finishers, and gilt development sites) belonging to 21 different swine production systems, denoted as numbers 1–22 (no system 3) in 10 states of the US. Three isolates originated from a production system in Mexico (labeled MEX). Isolates were stored at -80°C until further testing, for which they were re-grown in 3 mL aliquots of modified Hayflick’s medium for 2 to 14 days [26]. The suspension was centrifuged, and *M*. *hyorhinis* genomic DNA was extracted using the DNeasy Blood & Tissue kit following the manufacturer’s protocol (Qiagen, Germantown, MD).

### Loci selection and primer design

The whole genomes of four *M*. *hyorhinis* isolates available in GenBank (HUB-1 (NC_014448.1), GDL-1 (NC_016829.1) MCLD (NC_017519.1) and SK76 (NC_019552.1)) were utilized to identify potential target genes [27–30]. Identification of variable regions within the *M*. *hyorhinis* species was accomplished through a Clustal W progressive alignment of all 4 *M*. *hyorhinis* genomes using Mauve 2.3.1[31]. Further identification of areas of variation within different housekeeping and surface protein encoding genes, such as previously identified variable lipoproteins and adhesins [32], was performed using Geneious 7.0.6 (Biomatters, Auckland, New Zealand, http://www.geneious.com). Primers were designed using PrimerQuest® (IDT, Coralville, USA) software. In silico analysis of primer specificity was carried out using the Primer-BLAST tool (https://www.ncbi.nlm.nih.gov/tools/primer-blast). Genes with observed genetic variation between the available *M*. *hyorhinis* genomes were further tested with a subset of 6 distinct isolates originating in pigs from different production systems, ages, and geographical locations. Candidate genes were discarded if no nucleotide sequence variation was observed between these 6 isolates.

### PCR conditions and sequencing

The loci selected were amplified in an Eppendorf thermal cycler (Eppendorf Mastercycler 5345, Hamburg, Germany) with a total volume of 25 μl. The reaction mixture contained 12 μl of HotStarTaq Master Mix (Qiagen, Germantown, MD), 0.8 μl of each primer (1 μM), 9.4 μl of H2O, and 2 μl of template DNA. PCR conditions were: 95°C for 15 min, followed by 35 cycles of 95°C for 1 min, 43.5°C for 1:30 mins, and 72°C for 1 min, and a final extension of 72°C for 10 min. PCR products were analyzed by electrophoresis using an 1.5% agarose gels stained with ethidium bromide, run at 120 V for 40 min, and visualized using a gel imaging system. PCR products were purified using the QIAquick PCR Purification Kit (Qiagen, Germantown, MD) and bi-directionally sequenced by standard Sanger sequencing on an ABI 3730xl BigDye Terminator v 3.1 (Life Technologies, Grand Island, NY).

### Data analysis

Resulting sequencing data was quality assessed, and sequences were aligned utilizing ClustalW within Geneious 7.0.6 and trimmed to equal sizes. MEGA 5.2.1 (version 5; www.megasoftware.net) was utilized to identify parsimony-informative sites. Nucleotide sequence alignments for each gene fragment were translated to assess the quality of change (synonymous or non-synonymous substitutions). Individual and concatenated dendrograms were computed using the Hasegawa-Kishino-Yano distance model and the UPGMA (unweighted pair group method with arithmetic mean) tree building method.

For each targeted locus, each distinct sequence variant was arbitrarily assigned a consecutive number starting with 1, with no weight given to the amount of nucleotide sequence variation among alleles [23]. A distinct sequence type (ST) or allelic profile number was defined for each combination of the allele numbers for each locus (i.e. 1-5-2-3). The STs were assigned consecutive numbers in order of description (i.e. ST1). Relationships among strains were evaluated using minimum spanning trees (MST) analysis, after the introduction of allelic profiles into Bionumerics software V7.1 (Applied Math, Sint Maartens-Latem, Belgium). The strains were grouped into clonal complexes, defined here “as a group of MLST genotypes in which every genotype shares at least 3 out of 4 loci in common with at least one other member of the group” (pubmlst.org/analysis/burst/burst.shtml).

## Results

### Selection and evaluation of *M*. *hyorhinis* MLST-s marker genes

A total of 27 genes were evaluated as potential target genes (S2 Table). All housekeeping genes except *pdhB* and *ung* were nearly identical (~ 99.5%) in all 4 annotated genomes and were therefore not included for further testing. While variation was observed within the *nrdf* and *lspA* surface protein genes when comparing the 4 annotated genomes, testing of the initial 6 *M*. *hyorhinis* isolates revealed no nucleotide sequence variation and were therefore not included for further testing. The gene sequences for *vlpF* and *B* genes were found to be highly divergent among the 4 available genomes, with no conserved flanking regions, hindering primer placement. Primers for the remaining *vlp* genes were designed; however, an amplicon was not obtained for *vlpA*, *C*, *D* and *E* genes. Although an amplicon was generated for the *vlpG* gene, the generated product had low quality and was not reproducible.

Minimal nucleotide sequence variation was observed within the *cls*, *hexo* and *p37* genes and were therefore excluded from the subsequent testing of the remaining isolates. The discriminatory power of the technique was not affected after the removal of the 3 genes. Poor sequencing quality was obtained for the *p3* gene and thus was excluded from further analysis. The final MLST-s protocol included: *pdhB*, *p95*, *mtlD* and *ung* (Table 1). The selected genes were dispersed throughout the *M*. *hyorhinis* genome. In silico analysis revealed no cross-reactivity between primer sequences and swine related non-targeted taxa.

**Table 1:** Primer sequences employed in MLST-s *M*. *hyorhinis* assay.

Target gene	Gene name	Forward (5’-3’)	Reverse (5’-3’)
*pdhB*	Pyruvate dehydrogenase-E beta-1 subunit	AACGACCCAGATCCAGTTATTTT	TCGTTAATTGCGTGGAATCCTTC
*p95*	Outer membrane protein p95	GTTGCCAAGCAAGATGCTAAA	TGCTGAATGTACTCACCTGAAA
*mtlD*	Mannitol-1-phosphate 5-dehydrogenase	GGTGCAGGTAGCATTGGTCGTGG	GCCAAGATAACCCAAATAAGAATGC
*ung*	Uracil-dna glycosylase	GCAAGAACAAGACAAAGAAT	TAGCTAAACGGTGAAGGATGAGATA

### Relatedness of *M*. *hyorhinis* isolates

The dendrogram constructed based upon concatenated gene sequences (1441bp) revealed genetic polymorphisms among the examined isolates (S1 Fig). The nucleotide sequence variation (percent informative sites) within each gene ranged from 1.4% to 21% (Table 2), with *mtlD* showing the highest degree of variation and *pdhB* the lowest. Although nucleotide polymorphisms were observed, the majority resulted in synonymous changes in the *pdhB*, *p95* and *ung* genes. In contrast, non-synonymous changes were seen within the *mtlD* gene (S2 Fig). Furthermore, in some instances, changes involved the switch from amino acids belonging to different groups, based on the characteristics of the side chain, polarity, or acid-base properties (i.e. alanine to serine, glutamic acid to lysine) (S2 Fig). The number of alleles per gene varied from six to 20, giving rise to 39 sequence types (STs) within the 104 isolates (Tables 2 and 3).

**Table 2:** Characteristics of regions used for multilocus sequence analysis and number of alleles.

Target gene	Size (bp)	Trimmed length (bp)	Percent evaluated	Variable sites^a^	Parsimony-informative sites^b^	Percent informative sites (%)	No. of alleles
*pdhB*	987	415	42	6	6	1.4	7
*p95*	3171	289	9	8	6	2	9
*mtlD*	1101	537	49	120	115	21	20
*ung*	675	200	30	4	4	2	6

^a^ A variable site contains at least two types of nucleotides or amino acids.

^b^A site is parsimony-informative if it contains at least two types of nucleotides (or amino acids), and at least two of them occur with a minimum frequency of two.

**Table 3:** Frequency and distribution of 39 *M*. *hyorhinis* STs within sample type, system, origin, and age of pig.

ST	ST profile*	Frequency	Clonal complex	Sample type (no)^†^	System^‡^	State of origin	Age (weeks)
1	1-1-1-1	3	1	Pericardium	1, 21	MN	7,13
2	1-1-1-2	1	1	Pericardium	4	NC	3
3	1-1-1-3	2	1	Pericardium, pleura	8	MN	7
4	1-1-2-2	2	1	Pleura, joint	13, 14	TN, IL	7,9
5	1-1-2-3	1	1	Pericardium	8	IA	9
6	1-1-3-2	1	1	Bronchus	9	NE	13
7	1-1-8-2	2	1	Joint	13	IL	9
8	1-1-11-2	1	1	Pleura	17	NC	NA
9	1-1-16-2	1	1	Bronchus	6	NE	5
10	1-1-17-2	3	1	Peritoneum (1), bronchus (2)	5, 22	OK, NE	4,11
11	1-3-1-1	1	1	Joint	12	MN	8
12	1-3-1-2	1	1	Pericardium	7	MN	11
13	1-3-5-4	1	S	Pleura	11	NC	10
14	1-4-3-2	1	1	Pleura	9	NE	13
15	1-5-1-1	4	1	Bronchus (1), pericardial (2), nasal (1)	2	MN	8,11,13
16	1-5-1-2	1	1	Joint	2	MN	7
17	1-5-1-4	9	1	Bronchus (3), joint (1), pericardium (2), pleura (3)	5,7,10, 15,18	KS,MN,IN, OK	7,8,9,11,13,50
18	1-5-4-1	5	1	Bronchus(3), joint (1), nasal (1)	2	MN	8,13
19	1-5-9-1	9	1	Bronchus (2), joint (3) nasal (4)	2	MN	6,8,13
20	1-5-13-1	1	1	Pleura	2	MN	8
21	1-6-10-2	1	S	Pleura	16	PA	4
22	1-7-18-1	1	S	Pericardium	19	MEXICO	20
23	1-8-1-1	1	1	Pleura	12	MN	5
24	1-8-15-2	1	2	Pericardium	19	MEXICO	8
25	1-8-19-2	1	2	Pericardium	19	MEXICO	10
26	2-1-1-2	1	1	Nasal	REF^§^	IA	NA
27	2-2-2-2	4	3	Pleura	5	KS, OK	3,6
28	2-2-7-2	2	3	Joint	NA	NA	NA
29	3-5-1-4	5	1	Bronchus, pericardium, pleura	5	OK	8,10
30	3-5-14-1	2	S	Aerosol, bronchus	8	MN	6
31	4-3-14-5	5	S	Pericardium	5	OK	8
32	4-5-15-2	6	S	Nasal cavity, pleura	5	OK	4,5
33	5-5-4-1	1	1	Nasal	2	MN	8
34	5-5-4-2	1	1	Pericardium	2	MN	6
35	5-5-9-1	8	1	Bronchus (2), joint (3), pericardial (2), pleura (1)	2	MN	8
36	6-1-6-2	2	S	Joint	NA	NA	NA
37	7-3-12-2	13	S	Bronchus (8), nasal cavity (3), pericardium (2)	7	MN	14
38	7-8-3-2	1	S	Pericardium	20	MN	10
39	7-9-20-6	1	S	Nasal	HUB-1^¦^	CHINA	NA

* Order of allele number for each locus: *pdhB*, *p95*, *mtlD* and *ung*

^*†*^ Type of sample where isolate was obtained

^*‡*^ Systems are defined as the same owner and denoted as a number from 1–22

NA = not available

S = singleton, not part of a clonal complex

^§^ Reference strain (ATCC 17981)

^¦^ HUB-1 reference genome (GenBank)

### Frequency and distribution of *M*. *hyorhinis* sequence types (ST)

**Fig 1:**
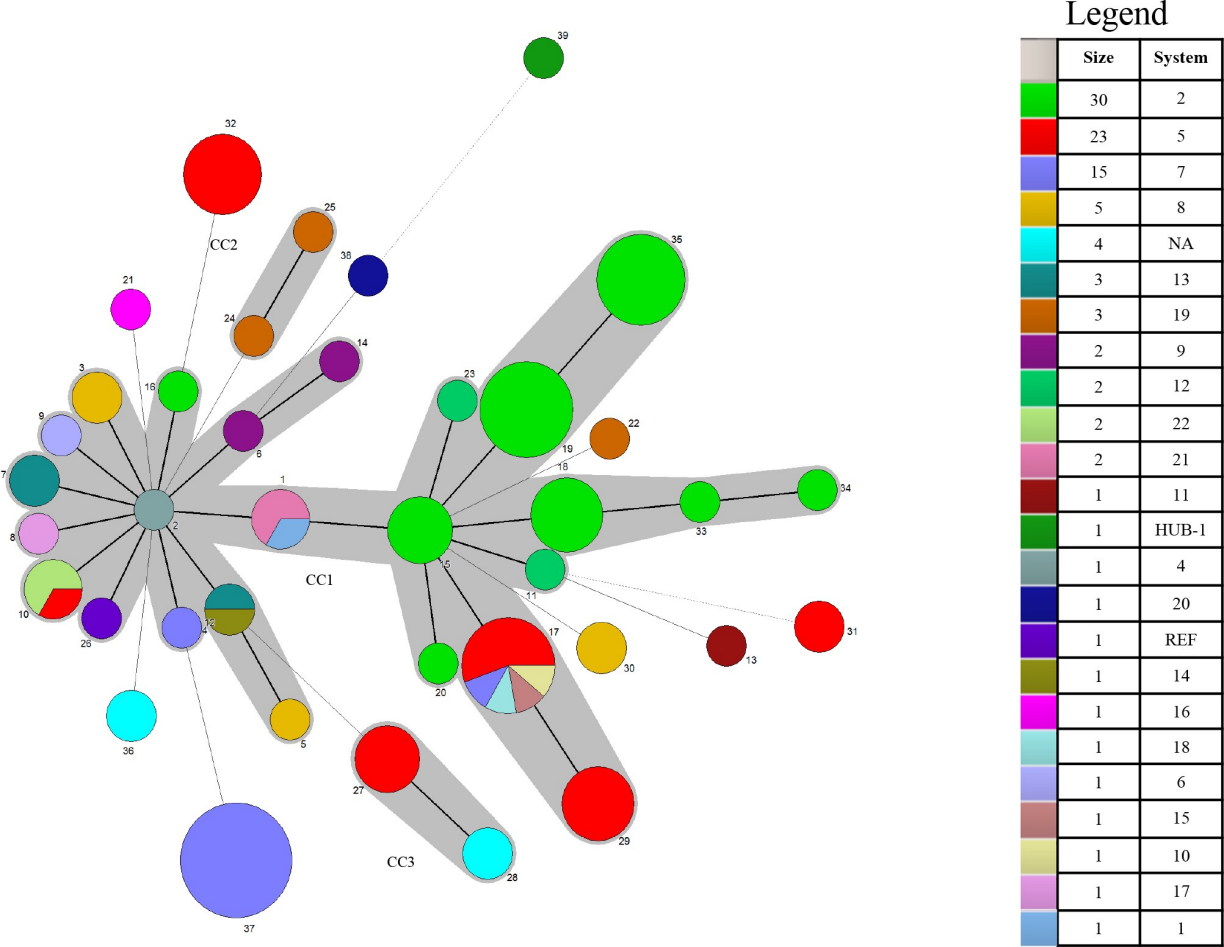
Minimum spanning tree analysis for 104 *M*. *hyorhinis* isolates. Each circle corresponds to a sequence type (ST). Size of the circle represents numbers of isolates with same ST. Lines between STs indicate inferred phylogenetic relationships. Thickness of the line represents the number of allelic mismatches between the STs (bold = 1, plain = 2, dotted = 3). Grey zones that surround STs belong to the same clonal complex (CC). Number near circles indicates ST number. Color of the circle indicates the swine production system. Legend: “size” indicates number of isolates and “system” indicates system number.

The most frequent ST, ST37, was observed 13 times. Eighteen STs contained between 2 and 9 isolates, and 21 STs contained only one isolate. Minimum spanning tree analysis showed the grouping of 39 STs into 3 clonal complexes (CC), with CC1 encompassing more STs (n = 25) and isolates (n = 66) than any other clonal complex. CC2 and CC3 contained 2 STs each and a total of 2, and 6 isolates, respectively. Lastly, singletons, defined as isolates which share less than 3 out of 4 loci with another member of a clonal complex, were identified 10 times and included 30 isolates (Fig 1).

**Fig 2:**
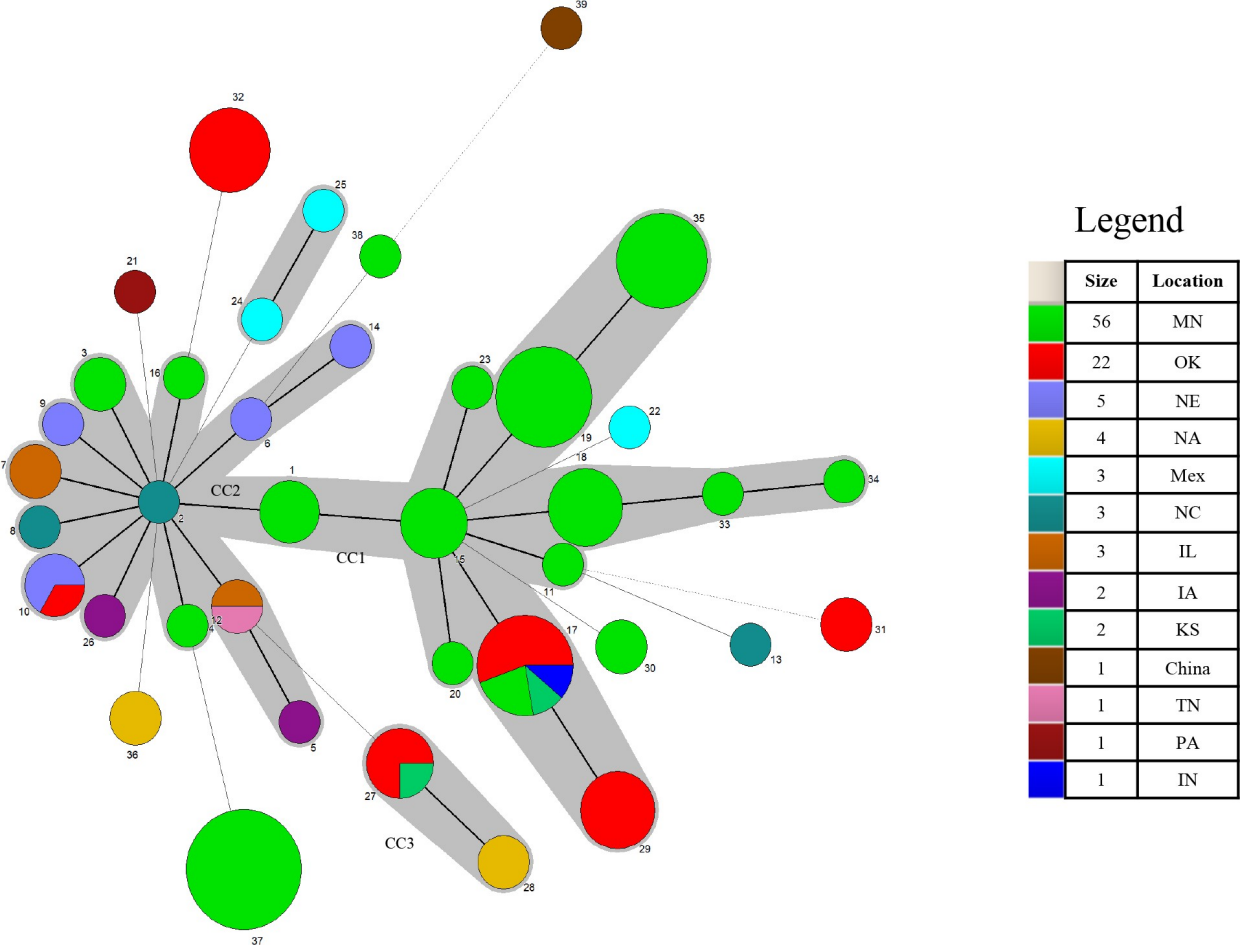
Minimum spanning tree analysis for 104 *M*. *hyorhinis* isolates. Each circle corresponds to a sequence type (ST). Size of the circle represents numbers of isolates with same ST. Lines between STs indicate inferred phylogenetic relationships. Thickness of the line represents the number of allelic mismatches between the STs (bold = 1, plain = 2, dotted = 3). Grey zones that surround STs belong to the same clonal complex (CC). Number near circles indicates ST number. Color of circle represents the geographical location. Legend: “size” indicates number of isolates and “location” indicates the geographical location of the ST.

**Fig 3:**
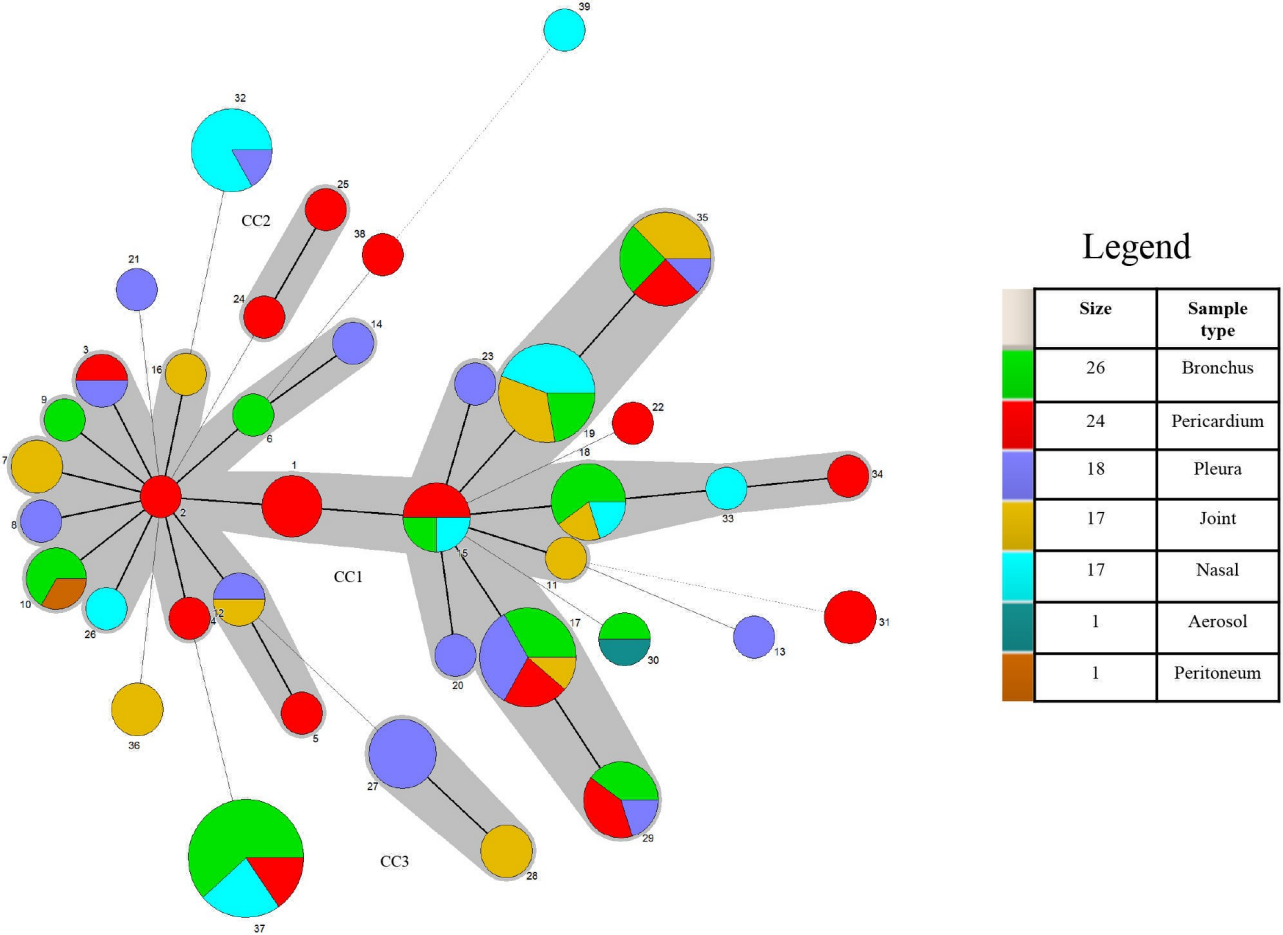
Minimum spanning tree analysis for 104 *M*. *hyorhinis* isolates. Each circle corresponds to a sequence type (ST). Size of the circle represents numbers of isolates with same ST. Lines between STs indicate inferred phylogenetic relationships. Thickness of the line represents the number of allelic mismatches between the STs (bold = 1, plain = 2, dotted = 3). Grey zones that surround STs belong to the same clonal complex (CC). Number near circles indicates ST number. Color of circle represents type of sample. Legend: “size” indicates number of isolates and “sample type” indicates the anatomical location where the ST originated.

ST37, the most frequent subtype, and ST19 contained isolates from only one management system (system 7 and 2, respectively), where specimens were collected the same day during an *M*. *hyorhinis* outbreak investigation (Table 3). Furthermore, two isolates from the upper respiratory tract (nasal cavity or bronchus) and pericardium were obtained from the same pig, evidencing that isolates recovered from systemic sites of diseased pigs can be also found in the nasal cavity or bronchi. ST19 was represented by isolates of healthy pigs from the nasal cavity or bronchi and diseased pigs from joints originating from system 2 in 2010 through 2012. Isolates with different STs (ST18 and ST35), however, were also obtained from pigs from that system during the same time frame (Table 3). Furthermore, in system 2, isolates from the pleura and bronchus of the same pig had different STs, differing by only one allele (*mltD*). In system 5, isolates in different U.S. states shared the same ST, suggesting a point source introduction. In contrast, 5 different STs were identified within the same system (Figs 1,2 and 3).

The isolates from ST17 (n = 9) originated from systemic sites of pigs of wide age distribution (7–50 weeks), and from 5 different systems in 4 different states collected in 2010–2012 (Table 3). It is important to note that information gathered for each isolate included “system,” which refers to the ownership of the pigs but lacks information on the pig flow or site. Therefore, the epidemiological interpretation of the data presented here may be limited since one system can be comprised of multiple producers who submit cases independently but share the same origin of pigs.

The reference strain HUB-1, originating from China, as well as the strains originating from Mexico showed unique STs. CC2 was comprised of isolates from the same system in Mexico (Table 3), while CC3 included isolates from different systems. Finally, no evident relationships were observed with respect to the age of the pig, lesion type, year, or US state of origin (Figs 1,2 and 3). However, strains originating from different countries (US, China and Mexico) clustered in distinct clonal complexes or as singletons. Additionally, there was no evidence of a specific ST being associated with virulence.

## Discussion

The objective of this study was to develop an MLST-s typing scheme for epidemiological and genetic characterization of *M*. *hyorhinis* field isolates. The novel scheme was utilized on a set of 104 well-characterized isolates from various tissue types, swine production systems, ages and geographic locations. Twenty-seven loci were evaluated as potential gene targets; however, many of these loci were almost identical in 4 previously published *M*. *hyorhinis* genomes and therefore not pursued further. A previously published and later optimized MLST scheme, showed an overall low percentage of polymorphic sites within each of the included housekeeping genes [21, 22]. In view of the limited genetic variation observed in housekeeping genes, more variable genes, such as surface protein encoding genes, were incorporated. This modification could increase the possibility of detecting more genetic variation, making it useful for typing during localized disease outbreaks and studying the pathogenesis of *M*. *hyorhinis* [24]. In contrast, variable lipoprotein encoding genes revealed an immense amount of variation between the available genomes, lacking conserved areas for primer placing. These findings were somewhat anticipated given the fact that the genes coding for these lipoproteins are under constant change, either through recombination or high-frequency mutations, which affects their expression and size [33, 34].

The final MLST-s protocol contained 2 core housekeeping genes, *pdhB* and *ung*, with an observed variation between 1.4–2% resulting in 6 and 7 different allele types, respectively. Similar amounts of variation have also been observed within housekeeping genes in previously published MLST schemes for *M*. *hyopneumoniae*, *M*. *agalactiae*, *M*. *bovis and M*. *hyorhinis* [20–22, 35, 36]. One unanticipated finding was the little to no variation observed within widely recognized adhesins, such as *p37* and *p95*. The recent sequencing of the complete p37 gene in 33 unrelated isolates from France showed 18 different nucleotide sequences.[21] Although variation was observed, the previous study concluded that the discriminatory power was relatively poor and that single-gene typing methods can fail at differentiating isolates that have a reduced variation. One possible explanation for the lack of variation is that the proteins encoded by these genes may be essential for host colonization and survivability [37]. In fact, it has been shown that truncated versions of certain adhesins within another swine mycoplasma species, *M*. *hyopneumoniae*, contribute to loss of virulence [38].

Even though variation was observed within *pdhB*, *p95* and the *ung* loci, these nucleotide changes resulted in mostly synonymous changes. These findings are in accordance with previous results, where the genetic variation observed in housekeeping genes generally led to synonymous changes [21]. On the other hand, the surface protein-encoding gene *mtlD* showed great variation (~ 21%) within the analyzed sequences and more than 20 different alleles. The predicted amino acid sequence alignment of *mtlD* revealed multiple non-synonymous changes. In some cases, the changes were between amino acids of different chemical groups. Future studies should evaluate if these changes influence the antigenic variation of this species due to the protein location on the bacterial surface or if the changes contribute to virulence.

Modified MLST protocols are not novel. Hyper-variable genes have been incorporated into typing schemes for *M*. *agalactiae*, *M*. *capricolum* and *B*. *pertussis* [35, 39, 40]. In all cases, an increase in discriminatory power was obtained; in some cases, this modification led to the identification of MLST profiles associated with epidemics and globally distributed strains [40]. The increased ability to discriminate between isolates during an epidemic or outbreak investigation might be at the cost of the interpretation of observed evolutionary relationships [24]. In this study, most systems had at least two STs within a 2-year period, and most frequently the strains differed at the *mtlD* loci, evidencing that the addition of hyper-variable genes might be a useful tool for local epidemiological investigations.

The present study showed high genetic variation within the *M*. *hyorhinis* species, with multiple STs circulating within US swine herds. The MLST-s scheme presented here was revealed to be highly discriminatory, capable of subdividing 104 isolates into 39 distinct STs. Multiple STs were found within a single system and an individual pig during the same time frame. Yet, frequently, isolates from the same farm shared the same ST. ST17 included isolates from 5 different production systems. This result could indicate the dissemination of a clone that may be a more successful pathogen, the existence of an organism with a longer persistence and survivability, or a common gilt source between the systems. It could also be due to the convenient nature of the sampling performed here [35]. In contrast to the observed sharing of ST17 between systems, the majority of STs with more than one isolate originated from the same system.

No correlation between STs and the age of the pig, year of isolation, sample type, pathogenicity, and state of US origin was observed. These observations are in accordance with others [22,41]. However, in this study three distinct clonal complexes, two of which included strains from the US and one that exclusively contained strains from Mexico, were identified. This is in contrast with the findings generated with the previously published MLST scheme, where only one clonal complex was identified in each data set, that included isolates from three different countries [21, 22]. This finding suggests that the novel MLST-s presented here could have a higher discriminatory power compared to previously published schemes, possibly due to the addition of more variable genes. However, this study lacked a side by side comparison and therefore further research is required to test such hypothesis. Finally, it is possible that the over-representation of isolates in this dataset from diseased pigs could have hindered the identification of virulence-associated STs.

Currently, swine practitioners are faced with the challenge of controlling *M*. *hyorhinis* disease in affected post-weaning pigs. The application of the MLST-s protocol described here has resulted in better understanding of the diversity of *M*. *hyorhinis* field isolates circulating in US swine herds. This tool could help to elucidate further features of the epidemiology and dynamics of infection for this pathogen, understand disease outbreaks, assist with selection of isolates for vaccine production, and identify the potential origin of a specific isolate.

## Supporting information

S1 Table Epidemiological characteristics of the 104 *M*. *hyorhinis* isolates. (DOCX)

S2 Table Description and location of the 27 genes evaluated. (DOCX)

S1 Fig Inferred relationships between 104 *M*. *hyorhinis* isolates using concatenated sequences for genes: *pdhB*, *p95*, *mtlD* and *ung*. Data was analyzed in Geneious using the UPGMA tree method based on the Hasegawa-Kishino-Yano model. A total of 1,441 positions were employed in the final dataset and 1500 bootstrap replicates. Scale represents nucleotide substitutions per site.
(TIF)

S2 Fig Amino acid sequence alignment for the *mtlD* gene. Arrows indicate areas of non-synonymous changes between aligned sequences where the amino acid change was between different amino acid groups, based on polarity, pH level or side chain. Arrows from left to right: lysine (basic) vs. glutamic acid (acid), alanine (non-polar) vs. serine (polar) phenylalanine (aliphatic group) vs. leucine (aromatic).
(PNG)

S1 Data Concatenated gene (*pdhB*, *p95*, *mtlD* and *ung*) sequences of all 104 *M*. *hyorhinis* isolates. (CSV)

## References

[1] Rovira A, Clavijo MJ, Oliveira S. *Mycoplasma hyorhinis* infection of pigs. Acta Sci Vet. 2010;38(Suppl 1): s9–15.

[2] Clavijo MJ, Murray D, Oliveira S, Rovira A. Infection dynamics of *Mycoplasma hyorhinis* in three commercial pig populations. Vet Rec. 2017 7 15;18(3): 68 10.1136/vr.104064 28424318

[3] Kobisch M, Friis NF. Swine mycoplasmoses. Rev Sci Tech. 1996 12;15(4): 1569–605. 10.20506/rst.15.4.983 9190026

[4] Neto JG, Gauger PC, Strait EL, Boyes N, Madson DM, Schwartz KJ. Mycoplasma-associated arthritis: critical points for diagnosis. J Swine Health Prod. 2012 3 1;20(2): 82–6.

[5] Goiš M, Pospíšil Z, Černy M, Mrva V. Production of pneumonia after intranasal inoculation of gnotobiotic piglets with three strains of *Mycoplasma hyorhinis*. J Comp Pathol. 1971 7 1;81(3): 401–10. 10.1016/0021-9975(71)90028-4 4935555

[6] Friis NF. A serologic variant of *Mycoplasma hyorhinis* recovered from the conjunctiva of swine. Acta Veterinaria Scandinavica. 1976;17(3): 343–53. 983893 10.1186/BF03547914 PMC8383955

[7] Friis NF. Mycoplasmas cultivated from the respiratory tract of Danish pigs. Acta Veterinaria Scandinavica. 1971;12(1): 69–79. 5575144

[8] Rosengarten R, Wise KS. Phenotypic switching in mycoplasmas: phase variation of diverse surface lipoproteins. Science. 1990 1 19;247(4940): 315–8. 10.1126/science.1688663 1688663

[9] Schulman A, Estola T, Garry-Andersson AS. On the occurrence of *Mycoplasma hyorhinis* in the respiratory organs of pigs, with special reference to enzootic pneumonia. Zentralbl Veterinarmed B. 1970 5;17(5): 549–53. 10.1111/j.1439-0450.1970.tb01565.x 5512059

[10] Goiš M, Kuksa F. Intranasal infection of gnotobiotic piglets with *Mycoplasma hyorhinis*: differences in virulence of the strains and influence of age on the development of infection. Zoonoses Public Health. 1974 6 1;21(5): 352–61. 10.1111/j.1439-0450.1974.tb00510.x 4844322

[11] Kinne J, Johannsen U, Neumann R, Mehlhorn G, Pfützner H. The pathology and pathogenesis of experimental *Mycoplasma hyorhinis* infection of piglets with and without thermomotoric stress. 1. Pathologic-anatomic, histologic and immunomorphologic study results. Zentralbl Veterinarmed A. 1991 5;38(4): 306–20. 1907790

[12] Clavijo MJ, Davies P, Morrison R, Bruner L, Olson S, Rosey E, et al Temporal patterns of colonization and infection with *Mycoplasma hyorhinis* in two swine production systems in the US. Vet Microbiol. 2019 7 234 p 110–118. 10.1016/j.vetmic.2019.05.021 31213266

[13] Zadoks RN, Schukken YH. Use of molecular epidemiology in veterinary practice. Vet Clin North Am Food Anim Pract. 2006 3 1;22(1): 229–61. 10.1016/j.cvfa.2005.11.005 16517304 PMC7126525

[14] Tankouo-Sandjong B, Sessitsch A, Liebana E, Kornschober C, Allerberger F, Hächler H, et al MLST-v, multilocus sequence typing based on virulence genes, for molecular typing of *Salmonella enterica subsp*. *enterica* serovars. J Microbiol Methods. 2007 4 30;69(1): 23–36. 10.1016/j.mimet.2006.11.013 17208323

[15] Barlev NA, Borchsenius SN. Continuous distribution of *Mycoplasma* genome 443 sizes. Biomed. Sci. 1991;2: 641–645. 1841633

[16] Yamaguti M, Oliveira RC, Marques LM, Buzinhani M, Buim MR, Neto RL, et al Molecular characterization of *Mycoplasma hyorhinis* isolated from pigs using pulsed-field gel electrophoresis and 16S rRNA sequencing. Vet Record Open. 2015 11 30;2(2): e000093 10.1136/vetreco-2014-000093 PMC4680736 26688737

[17] Tenover FC, Arbeit RD, Goering RV, Mickelsen PA, Murray BE, Persing DH, et al Interpreting chromosomal DNA restriction patterns produced by pulsed-field gel electrophoresis: criteria for bacterial strain typing. J Clin Microbiol. 1995 9;33(9): 2233–9. 7494007 10.1128/jcm.33.9.2233-2239.1995 PMC228385

[18] King SJ, Leigh JA, Heath PJ, Luque I, Tarradas C, Dowson CG, et al Development of a multilocus sequence typing scheme for the pig pathogen *Streptococcus suis*: identification of virulent clones and potential capsular serotype exchange. J Clin Microbiol. 2002 10 1;40(10): 3671–80. 10.1128/JCM.40.10.3671-3680.2002 12354864 PMC130843

[19] Olvera A, Cerda-Cuellar M, Aragon V. Study of the population structure of *Haemophilus parasuis* by multilocus sequence typing. Microbiology. 2006 12 1;152(Pt 12): 3683–90. 10.1099/mic.0.29254-0 17159221

[20] Mayor D, Jores J, Korczak BM, Kuhnert P. Multilocus sequence typing (MLST) of *Mycoplasma hyopneumoniae*: a diverse pathogen with limited clonality. Vet Microbiol. 2008 2 5;127(1–2): 63–72. 10.1016/j.vetmic.2007.08.010 17884308

[21] Tocqueville V, Ferré S, Nguyen NH, Kempf I, Marois-Créhan C. Multilocus sequence typing of *Mycoplasma hyorhinis* strains identified by a real-time TaqMan PCR assay. J Clin Microbiol. 2014 5 1;52(5): 1664–71. 10.1128/JCM.03437-13 24622092 PMC3993664

[22] Trüeb B, Catelli E, Luehrs A, Nathues H, Kuhnert P. Genetic variability and limited clonality of *Mycoplasma hyorhinis* in pig herds. Vet Microbiol. 2016 8 15;191: 9–14. 10.1016/j.vetmic.2016.05.015 27374901

[23] Maiden MC, Bygraves JA, Feil E, Morelli G, Russell JE, Urwin R, et al Multilocus sequence typing: a portable approach to the identification of clones within populations of pathogenic microorganisms. Proc Natl Acad Sci U S A. 1998 3 17;95(6): 3140–5. 10.1073/pnas.95.6.3140 9501229 PMC19708

[24] Cooper JE, Feil EJ. Multilocus sequence typing–what is resolved? Trends Microbiol. 2004;12(8): 373–377. 10.1016/j.tim.2004.06.003 15276613

[25] Clavijo MJ, Oliveira S, Zimmerman J, Rendahl A, Rovira A. Field evaluation of a quantitative polymerase chain reaction assay for *Mycoplasma hyorhinis*. J Vet Diagn Invest. 2014 11;26(6): 755–60. 10.1177/1040638714555175 25319032

[26] Lefèvre PC, Jones GE, Ojo MO. Pulmonary mycoplasmoses of small ruminants. Rev Sci Tech Off int Epiz. 1987;6(3): 713–757. 10.20506/rst.6.3.311 32370339

[27] Liu W, Fang L, Li S, Li Q, Zhou Z, Feng Z, et al Complete genome sequence of *Mycoplasma hyorhinis* strain HUB-1. J Bacteriol. 2010 11 1;192(21): 5844–5. 10.1128/JB.00946-10 20802032 PMC2953675

[28] Calcutt MJ, Foecking MF, Rosales RS, Ellis RJ, Nicholas RA. Genome sequence of *Mycoplasma hyorhinis* strain GDL-1. J Bacteriol. 2012 4;194(7): 1848 10.1128/JB.00033-12 22408248 PMC3302467

[29] Kornspan JD, Lysnyansky I, Kahan T, Herrmann R, Rottem S, Nir-Paz R. Genome analysis of a *Mycoplasma hyorhinis* strain derived from a primary human melanoma cell line. J Bacteriol. 2011 9 1;193(17): 4543–4. 10.1128/JB.05505-11 21705582 PMC3165497

[30] Goodison S, Urquidi V, Kumar D, Reyes L, Rosser CJ. Complete genome sequence of *Mycoplasma hyorhinis* strain SK76. Genome Announc. 2013 2 28;1(1). pii: e00101–12. 10.1128/genomeA.00101-12 23405353 PMC3569356

[31] Darling AE, Mau B, Perna NT. progressive Mauve: multiple genome alignment with gene gain, loss and rearrangement. PLoS One. 2010 6 25;5(6): e11147 10.1371/journal.pone.0011147 20593022 PMC2892488

[32] Siqueira FM, Thompson CE, Virginio VG, Gonchoroski T, Reolon L, Almeida LG, et al New insights on the biology of swine respiratory tract mycoplasmas from a comparative genome analysis. BMC genomics. 2013 3 14;14(1): 175. 23497205 10.1186/1471-2164-14-175 PMC3610235

[33] Citti C, Wise KS. *Mycoplasma hyorhinis* vlp gene transcription: critical role in phase variation and expression of surface lipoproteins. Mol Microbiol. 1995 11;18(4): 649–660. 10.1111/j.1365-2958.1995.mmi_18040649.x 8817488

[34] Citti C, Blanchard A. Mycoplasmas and their host: emerging and re-emerging minimal pathogens. Trends Microbiol. 2013 4;21(4): 196–203. 10.1016/j.tim.2013.01.003 23419218

[35] McAuliffe L, Gosney F, Hlusek M, De Garnica ML, Spergser J, Kargl M, et al Multilocus sequence typing of *Mycoplasma agalactiae*. J Medical Microbiol. 2011 6 1;60(Pt 6): 803–11. 10.1099/jmm.0.028159-0 21372188

[36] Manso-Silván L, Dupuy V, Lysnyansky I, Ozdemir U, Thiaucourt F. Phylogeny and molecular typing of *Mycoplasma agalactiae* and *Mycoplasma bovis* by multilocus sequencing. Vet Microbiol. 2012 12 28;161(1–2): 104–12. 10.1016/j.vetmic.2012.07.015 22841405

[37] Razin S. Peculiar properties of mycoplasmas: the smallest self-replicating prokaryotes. FEMS microbiology letters. 1992 12 15;100(1–3): 423–31. 10.1111/j.1574-6968.1992.tb14072.x 1478475

[38] Liu W, Xiao S, Li M, Guo S, Li S, Luo R, et al Comparative genomic analyses of *Mycoplasma hyopneumoniae* pathogenic 168 strain and its high-passaged attenuated strain. BMC genomics. 2013 12;14(1):80. 23384176 10.1186/1471-2164-14-80 PMC3626624

[39] Manso-Silván L, Dupuy V, Chu Y, Thiaucourt F. Multi-locus sequence analysis of *Mycoplasma capricolum subsp*. *capripneumoniae* for the molecular epidemiology of contagious caprine pleuropneumonia. Vet Res. 2011 7 14;42(1): 86 10.1186/1297-9716-42-86 21756321 PMC3177781

[40] van Loo IH, Heuvelman KJ, King AJ, Mooi FR. Multilocus sequence typing of *Bordetella pertussis* based on surface protein genes. J Clin Microbiol. 2002 6 1;40(6): 1994–2001. 10.1128/JCM.40.6.1994-2001.2002 12037054 PMC130760

[41] dos Santos LF, Clavijo MJ, Sreevatsan S, Rovira A, Moreira MA, Pieters M. Genotyping of *Mycoplasma hyorhinis* using multiple-locus variable number tandem repeat analysis. J Microbiol Methods. 2015 4 30;111: 87–92. 10.1016/j.mimet.2015.02.003 25661497

