# Supplementary material for: Molecular epidemiology of Mycoplasma hyorhinis porcine field isolates in the United States
Source: PLoS One. 2019 Oct 21;14(10):e0223653. doi: 10.1371/journal.pone.0223653 (PMC6802821; doi:10.1371/journal.pone.0223653)
Supplement: S1 Table — (DOCX) [file pone.0223653.s001.docx]

**S1 Table.** Epidemiological characteristics of the 104 *M. hyorhinis* isolates included in the study.

| Isolate ID | ST^a^ | Year | Sample type^b^ | System^c^ | State/country of origin | Age (weeks) |
| --- | --- | --- | --- | --- | --- | --- |
| 1 | 1 | 2011 | Pericardium | 1 | MN | 7 |
| 4 | 2 | 2011 | Pericardium | 4 | NC | 3 |
| 5 | 27 | 2011 | Pleura | 5 | KS | 3 |
| 6 | 26 | 1973 | Nasal | Reference strain | IA | NA |
| 7 | 9 | 2011 | Bronchus | 6 | NE | 5 |
| 8 | 12 | 2011 | Pericardium | 7 | MN | 11 |
| 9 | 3 | 2010 | Pericardium | 8 | MN | 7 |
| 10 | 6 | 2011 | Lung | 9 | NE | 13 |
| 11 | 14 | 2011 | Pleura | 9 | NE | 13 |
| 12 | 34 | 2011 | Pericardium | 2 | MN | 6 |
| 13 | 17 | 2011 | Joint | 10 | KS | 9 |
| 14 | 13 | 2011 | Pleura | 11 | NC | 10 |
| 15 | 36 | 2010 | Joint | NA | NA | NA |
| 16 | 28 | 2010 | Joint | NA | NA | NA |
| 17 | 28 | 2010 | Joint | NA | NA | NA |
| 18 | 36 | 2010 | Joint | NA | NA | NA |
| 19 | 27 | 2010 | Pleura | 5 | OK | 6 |
| 20 | 27 | 2010 | Pleura | 5 | OK | 6 |
| 21 | 11 | 2011 | Joint | 12 | MN | 8 |
| 22 | 7 | 2010 | Joint | 13 | IL | 9 |
| 23 | 4 | 2010 | Pleura | 14 | TN | 7 |
| 24 | 17 | 2010 | Pericardium | 15 | MN | 7 |
| 25 | 7 | 2010 | Joint | 13 | IL | 9 |
| 26 | 16 | 2010 | Joint | 2 | MN | 7 |
| 27 | 19 | 2010 | Joint | 2 | MN | 6 |
| 28 | 27 | 2010 | Pleura | 5 | OK | 6 |
| 30 | 3 | 2010 | Pleura | 8 | MN | 7 |
| 31 | 19 | 2010 | Joint | 2 | MN | 6 |
| 33 | 5 | 2010 | Pericardium | 8 | IA | 9 |
| 34 | 21 | 2010 | Pleura | 16 | PA | 4 |
| 35 | 4 | 2010 | Joint | 13 | IL | 9 |
| 38 | 8 | 2010 | Pleura | 17 | NC | NA |
| 39 | 17 | 2010 | Bronchus | 18 | IN | 7 |
| 40 | 19 | 2011 | Bronchus | 2 | MN | 8 |
| 41 | 35 | 2011 | Bronchus | 2 | MN | 8 |
| 42 | 15 | 2011 | Bronchus | 2 | MN | 8 |
| 43 | 18 | 2011 | Bronchus | 2 | MN | 8 |
| 44 | 35 | 2011 | Bronchus | 2 | MN | 8 |
| 45 | 33 | 2011 | Nasal | 2 | MN | 8 |
| 46 | 37 | 2011 | Bronchus | 7 | MN | 14 |
| 47 | 37 | 2011 | Nasal | 7 | MN | 14 |
| 48 | 37 | 2011 | Nasal | 7 | MN | 14 |
| 50 | 37 | 2011 | Nasal | 7 | MN | 14 |
| 51 | 37 | 2011 | Bronchus | 7 | MN | 14 |
| 52 | 37 | 2011 | Bronchus | 7 | MN | 14 |
| 54 | 10 | 2012 | Peritoneum | 5 | OK | 11 |
| 55 | 17 | 2012 | Pleura | 5 | OK | 11 |
| 56 | 22 | 2011 | Pericardium | 19 | MEXICO | 20 |
| 57 | 38 | 2011 | Pericardium | 20 | MN | 10 |
| 58 | 37 | 2011 | Bronchus | 7 | MN | 14 |
| 59 | 35 | 2011 | Pericardium | 2 | MN | 8 |
| 61 | 24 | 2011 | Pericardium | 19 | MEXICO | 8 |
| 62 | 25 | 2011 | Pericardium | 19 | MEXICO | 10 |
| 63 | 37 | 2011 | Bronchus | 7 | MN | 14 |
| 64 | 37 | 2011 | Bronchus | 7 | MN | 14 |
| 65 | 37 | 2011 | Bronchus | 7 | MN | 14 |
| 67 | 37 | 2011 | Pericardium | 7 | MN | 14 |
| 68 | 37 | 2011 | Bronchus | 7 | MN | 14 |
| 69 | 37 | 2011 | Pericardium | 7 | MN | 14 |
| 71 | 18 | 2011 | Bronchus | 2 | MN | 8 |
| 72 | 18 | 2011 | Joint | 2 | MN | 8 |
| 74 | 35 | 2011 | Pericardium | 2 | MN | 8 |
| 75 | 35 | 2011 | Joint | 2 | MN | 8 |
| 76 | 20 | 2011 | Pleura | 2 | MN | 8 |
| 77 | 35 | 2011 | Pleura | 2 | MN | 8 |
| 78 | 18 | 2011 | Bronchus | 2 | MN | 8 |
| 79 | 19 | 2011 | Bronchus | 2 | MN | 8 |
| 80 | 35 | 2011 | Bursa | 2 | MN | 8 |
| 81 | 19 | 2011 | Joint | 2 | MN | 8 |
| 82 | 35 | 2011 | Joint | 2 | MN | 8 |
| 83 | 15 | 2011 | Pericardium | 2 | MN | 8 |
| 85 | 1 | 2012 | Pericardium | 21 | MN | 13 |
| 86 | 1 | 2012 | Pericardium | 21 | MN | 13 |
| 87 | 29 | 2012 | Pericardium | 5 | OK | 10 |
| 88 | 17 | 2012 | Pericardium | 5 | OK | 11 |
| 89 | 29 | 2012 | Pericardium | 5 | OK | 10 |
| 90 | 29 | 2012 | Pleura | 5 | OK | 10 |
| 91 | 31 | 2012 | Pericardium | 5 | OK | 8 |
| 92 | 17 | 2012 | Pleura | 5 | OK | 11 |
| 93 | 23 | 2012 | Pleura | 12 | MN | 5 |
| 94 | 32 | 2012 | Pleura | 5 | OK | 4 |
| 95 | 15 | 2012 | Pericardium | 2 | MN | 11 |
| 96 | 17 | 2012 | Bronchus | 7 | MN | 50 |
| 98 | 17 | 2012 | Pleura | 5 | OK | 13 |
| 99 | 31 | 2012 | Pericardium | 5 | OK | 8 |
| 100 | 29 | 2012 | Bronchus | 5 | OK | 8 |
| 101 | 17 | 2012 | Bronchus | 5 | OK | 8 |
| 102 | 32 | 2012 | Nasal | 5 | OK | 4 |
| 103 | 15 | 2012 | Nasal | 2 | MN | 13 |
| 105 | 32 | 2012 | Nasal | 5 | OK | 4 |
| 106 | 32 | 2012 | Nasal | 5 | OK | 5 |
| 107 | 32 | 2012 | Nasal | 5 | OK | 4 |
| 108 | 32 | 2012 | Nasal | 5 | OK | 4 |
| 110 | 19 | 2012 | Nasal | 2 | MN | 13 |
| 111 | 10 | 2012 | Bronchus | 22 | NE | 4 |
| 113 | 18 | 2012 | Nasal | 2 | MN | 13 |
| 115 | 19 | 2012 | Nasal | 2 | MN | 13 |
| 116 | 19 | 2012 | Nasal | 2 | MN | 13 |
| 118 | 19 | 2012 | Nasal | 2 | MN | 13 |
| 122 | 10 | 2012 | Bronchus | 22 | NE | 4 |
| 124 | 30 | 2012 | Aerosol | 8 | MN | NA |
| 125 | 30 | 2012 | Bronchus | 8 | MN | 6 |
| 126 | 29 | 2012 | Bronchus | 5 | OK | 8 |
| 127 | 39 | NA | Nasal | Reference genome | CHINA | NA |

^a^ Sequence type from 1-39

*^b^* Type of sample where isolate was obtained

*^c^* Systems are defined as the same owner and denoted as a number from 1-22

NA- not available

^d^ Reference strain (ATCC 17981)

^e^ HUB-1 reference genome (GenBank)
