# Supplementary material for: Molecular epidemiology of Mycoplasma hyorhinis porcine field isolates in the United States
Source: PLoS One. 2019 Oct 21;14(10):e0223653. doi: 10.1371/journal.pone.0223653 (PMC6802821; doi:10.1371/journal.pone.0223653)
Supplement: S2 Table — (DOCX) [file pone.0223653.s002.docx]

**S2 Table.** Description and location of the 27 genes evaluated.

| Target gene | Description | Size (bp) | Location |
| --- | --- | --- | --- |
| fusA | Translation elongation factor G | 2088 | 618718-620805 |
| gyrB | DNA gyrase subunit B | 1923 | 315178-317100 |
| *pgmB* | Beta-phosphoglucomutase | 669 | 245172-245840 |
| lepA | GTP-binding protein | 1800 | 92319-94118 |
| *metS* | Methionine tRNA ligase | 1533 | 429598-431130 |
| *gltX* | Glutamyl-tRNA synthetase | 1446 | 414799-416244 |
| *dnaA* | Chromosomal replication initiator protein | 210 | 839406-839615 |
| *ung* | Uracil-dna glycosylase | 675 | 271562-272236 |
| *pdhB* | Pyruvate dehydrogenase E1 component beta subunit | 987 | 634129-635115 |
| *adk* | ATP AMP transphosphorylase | 669 | 488873-489541 |
| *gmk* | guanylate kinase | 579 | 522079-522657 |
| ropB | DNA-directed RNA polymerase subunit beta | 3711 | 69144-72854 |
| *P3* | Outer membrane protein p3 | 2184 | 531343-533526 |
| *p95* | Outer membrane protein p95 | 3171 | 539652-542822 |
| *P37* | Outer membrane protein p37 | 1212 | 766989-768200 |
| *mtlD* | Mannitol-1-phosphate 5-dehydrogenase | 1101 | 225414-226514 |
| *hexo* | hexosephosphate transport protein | 1449 | 527407-528855 |
| *nrdf* | Ribonucleoside-diphosphate reductase beta chai | 1035 | 817377-818411 |
| *lspA* | Lipoprotein signal peptidase II | 615 | 158456-159070 |
| *cls* | Cardiolipin synthetase | 1557 | 273452-275008 |
| *vlp A* | Variant surface antigen A | 1020 | 438653-439672 |
| *vlp B* | Variant surface antigen B | 705 | 441555-442259 |
| *vlp C* | Variant surface antigen C | 336 | 443110-443445 |
| *vlp D* | Variant surface antigen D | 480 | 433615-434094 |
| *vlp E* | Variant surface antigen E | 612 | 434404-435015 |
| *vlp F* | Variant surface antigen F | 201 | 435361-435561 |
| *vlp G* | Variant surface antigen G | 744 | 437529-438272 |
