# Supplementary figures and images for: Molecular epidemiology of Mycoplasma hyorhinis porcine field isolates in the United States
Source: PLoS One. 2019 Oct 21;14(10):e0223653. doi: 10.1371/journal.pone.0223653 (PMC6802821; doi:10.1371/journal.pone.0223653)

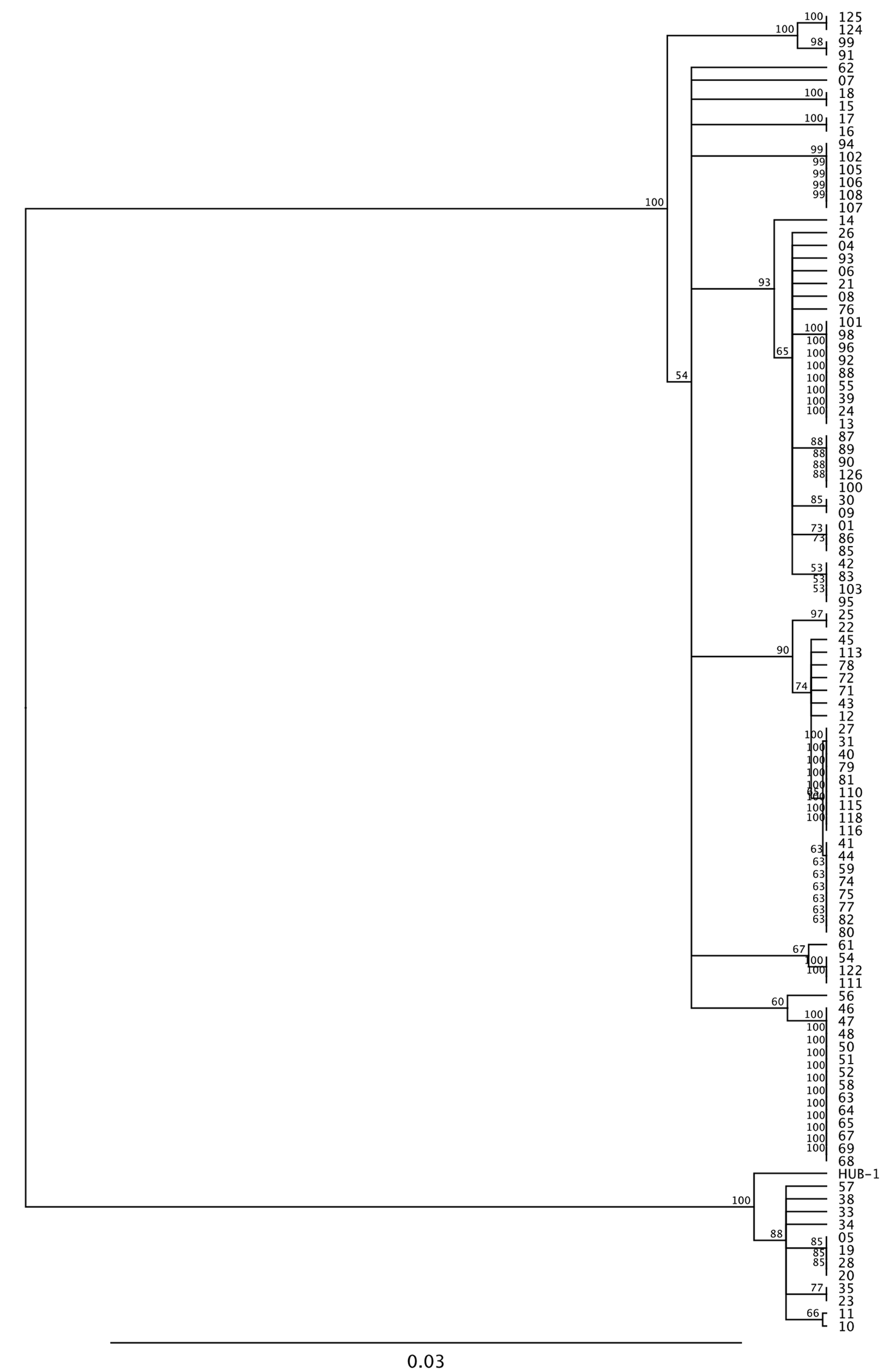

Supplement: S1 Fig — Data was analyzed in Geneious using the UPGMA tree method based on the Hasegawa-Kishino-Yano model. A total of 1,441 positions were employed in the final dataset and 1500 bootstrap replicates. Scale represents nucleotide substitutions per site. (TIF) [file pone.0223653.s003.tif]

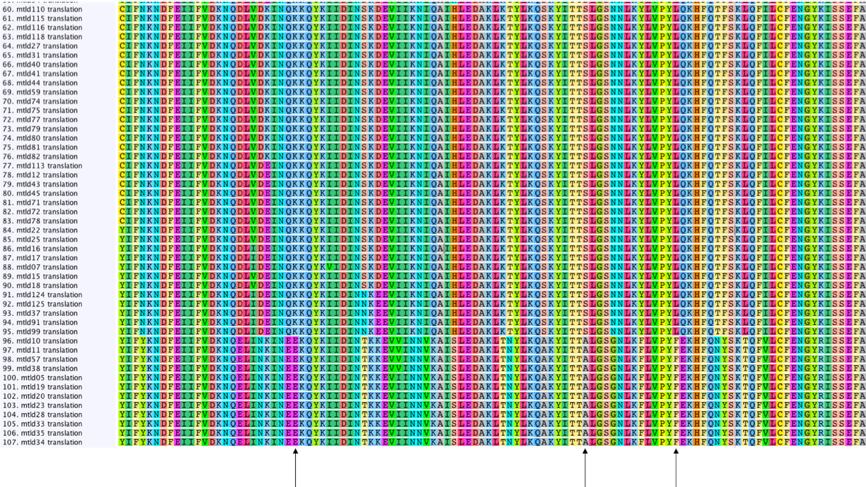

Supplement: S2 Fig — Arrows indicate areas of non-synonymous changes between aligned sequences where the amino acid change was between different amino acid groups, based on polarity, pH level or side chain. Arrows from left to right: lysine (basic) vs. glutamic acid (acid), alanine (non-polar) vs. serine (polar) phenylalanine (aliphatic group) vs. leucine (aromatic). (PNG) [file pone.0223653.s004.png]
